# A Mobile Phone Based Method to Assess Energy and Food Intake in Young Children: A Validation Study against the Doubly Labelled Water Method and 24 h Dietary Recalls

**DOI:** 10.3390/nu8010050

**Published:** 2016-01-15

**Authors:** Christine Delisle Nyström, Elisabet Forsum, Hanna Henriksson, Ylva Trolle-Lagerros, Christel Larsson, Ralph Maddison, Toomas Timpka, Marie Löf

**Affiliations:** 1 Department of Biosciences and Nutrition, Karolinska Institutet, NOVUM, Huddinge 141 83, Sweden; christine.delisle.nystrom@ki.se; 2 Department of Clinical and Experimental Medicine, Faculty of the Health Sciences, Linköping University, Linköping 581 83, Sweden; elisabet.forsum@liu.se (E.F.); hannahenriksson77@hotmail.se (H.H.); 3 Clinical Epidemiology Unit, Department of Medicine, Karolinska Institutet, Stockholm 171 77, Sweden; ylva.trolle@ki.se; 4 Department of Food and Nutrition, and Sport Science, University of Gothenburg, P.O. Box 300, Gothenburg 405 30, Sweden; christel.larsson@gu.se; 5 National Institute for Health Innovation, The University of Auckland, P.O. Box 92019, Auckland 1142, New Zealand; r.maddison@auckland.ac.nz; 6 Department of Medical and Health Sciences, Faculty of the Health Sciences, Linköping University, Linköping 581 83, Sweden; toomas.timpka@liu.se

**Keywords:** mobile phones, energy intake, food intake, total energy expenditure, child, DLW, 24 h dietary recall

## Abstract

Mobile phones are becoming important instruments for assessing diet and energy intake. We developed the Tool for Energy Balance in Children (TECH), which uses a mobile phone to assess energy and food intake in pre-school children. The aims of this study were: (a) to compare energy intake (EI) using TECH with total energy expenditure (TEE) measured via doubly labelled water (DLW); and (b) to compare intakes of fruits, vegetables, fruit juice, sweetened beverages, candy, ice cream, and bakery products using TECH with intakes acquired by 24 h dietary recalls. Participants were 39 healthy, Swedish children (5.5 ± 0.5 years) within the ongoing Mobile-based Intervention Intended to Stop Obesity in Preschoolers (MINISTOP) obesity prevention trial. Energy and food intakes were assessed during four days using TECH and 24 h telephone dietary recalls. Mean EI (TECH) was not statistically different from TEE (DLW) (5820 ± 820 kJ/24 h and 6040 ± 680kJ/24 h, respectively). No significant differences in the average food intakes using TECH and 24 h dietary recalls were found. All food intakes were correlated between TECH and the 24 h dietary recalls (*ρ* = 0.665–0.896, *p* < 0.001). In conclusion, TECH accurately estimated the average intakes of energy and selected foods and thus has the potential to be a useful tool for dietary studies in pre-school children, for example obesity prevention trials.

## 1. Introduction

Being overweight or obese in childhood is a global issue affecting around 42 million children under the age of five [1]. If the present trends persist, roughly 70 million young children will be classified as overweight or obese by 2025 [1]. Childhood obesity is of serious concern because it can persist throughout the lifespan, having various physical and psychological consequences [2]. One study found that children who were overweight during their pre-school years had a 60% risk of being overweight at 12 years of age [3] and another study reported that 34% of children classified as overweight at age seven were obese at age 13 [4]. Therefore, there is a need to target young children and conduct interventions in the pre-school years. There are few obesity prevention interventions targeting pre-school children [5], however interest has piqued in recent years [6,7,8,9,10].

In order to conduct such interventions, accurate and simple methods are needed to collect dietary data. To date, weighed or estimated food records and 24 h dietary recalls are commonly used to obtain dietary data, however these methods are burdensome on the participants and have limited accuracy [11,12]. New techniques that reduce participant burden, are easy to administer, and can be scaled are warranted. Two recent reviews of the literature suggested that participants favor dietary assessment using mobile phones over traditional methods [13,14]. However, the majority of mobile phone based dietary assessment methods have only been tested in pilot and feasibility studies in adults [14,15]. Therefore, there is a need to develop new methods for assessing diet and validate them in a variety of populations, such as young children.

We have developed the Tool for Energy Balance in Children (TECH) [16], a mobile phone dietary assessment method to measure energy and food intake in young children. TECH was created to be used in the study called Mobile-based intervention intended to stop obesity in preschoolers (MINISTOP), which is a population based randomized controlled trial that was initiated in 2014 in the county of Östergötland in Sweden [6,17]. Overall, the trial aims to determine the effectiveness of a 6-month mobile phone based intervention to improve body composition, dietary habits, physical activity, physical fitness and sedentary behavior in healthy pre-school aged children [6]. The specific aims of this nested validation study were: (a) to compare energy intake (EI) using TECH with total energy expenditure (TEE) measured via the doubly labelled water (DLW) method [18]; and (b) to compare the intakes of fruits, vegetables, fruit juice, sweetened beverages, candy, ice cream, and bakery products measured using TECH with intakes acquired by means of 24 h dietary recalls in pre-school aged children.

## 2. Materials and Methods

### 2.1. Participants and Study Design

Parents from the MINISTOP trial [6,17] were asked to participate with their child in this validation study, and were recruited at the final follow-up assessment (beginning in February 2015). A total of 45 parent child dyads were consecutively asked to participate until 40 agreed to do so. One parent child dyad did not submit food pictures and was excluded from the analysis, therefore the final sample comprised of 39 children (19 from the intervention group and 20 from the control group). Characteristics of the study sample in terms of age, weight, height, and BMI as well as the parental age, BMI, and education were comparable with those in the whole MINISTOP trial (*n* = 315). Parents were instructed to collect two urine samples from their child and bring them when they came for the final follow-up assessment at Linköping University Hospital. Weight and height of the children were recorded without shoes and with minimal clothing using an electronic scale and a wall stadiometer. Thereafter, the children were given a dose of stable isotopes mixed with fruit juice to measure their TEE for the following 14 days. The parents were instructed to collect urine samples on days 1, 5, 10 and 14 after dosing and to note the time of sampling. In the same 14 day period, intakes of foods and drinks were assessed using TECH and 24 h dietary recalls during the same four days. This study was approved by the Research and Ethics Committee, Stockholm, Sweden (2013/1607-31/5; 2013/2250-32) on the 10th of October 2013. MINISTOP is registered as a clinical trial (https://clinicaltrials.gov/show/NCT02021786).

### 2.2. Total Energy Expenditure

Each child was given an accurately weighed dose of stable isotopes using ^2^H_2_O (enrichment 99.9%) and H_2_^18^O (enrichment 20%): 0.14 g ^2^H_2_O and 0.35 g H_2_^18^O per kg of body weight. Urine samples were stored in glass vials with an internal aluminium-lined screw cap sealing at +4 °C until sample collection was finished, after which they were stored at −20 °C until analysis. ^2^H and ^18^O enrichments of dose and urine samples were analyzed (both pre and post dosing) using a Finnigan MAT Delta Plus Isotope-Ratio Mass Spectrometer (ThermoFinnigan, Gothenburg, Sweden) as described previously [19]. ^2^H dilution space (*N*_D_) and ^18^O dilution space (*N*_O_) were calculated using zero-time enrichments obtained from the exponential isotope disappearance curves that provided estimates for the elimination rates for ^2^H and ^18^O, respectively. N_D_/N_O_ was 1.039 ± 0.008 for the 39 children. CO_2_ production was calculated according to the method by Davies *et al.* [20] assuming that 27.1% of the total water losses were fractionated. This fractionation proportion was obtained by interpolation using values for children between 4.5 [20] and 7 years of age [21]. TEE was calculated from CO_2_ production using the Weir equation [22] assuming a food quotient of 0.85 [23].

### 2.3. Tool for Energy Balance in Children (TECH)

Parents were instructed to take pictures of every food and beverage consumed, excluding water for four separate days of their choice using their smartphone when they were home with their child. Two pictures from different angles were taken before and after the food or beverage was consumed. The parents were also instructed to provide additional pictures (before and after) if their child had another portion of food or drink. The parents were also asked to use both the provided standardized china (bowl, plate, and cup) and the checkered reference marker, in all pictures. The china and reference marker were used to give the nutritionists a better perspective when analyzing the pictures. To ensure that the parents took the pictures in a correct way they received both oral and written instructions on how to take the pictures. The written instructions included examples with pictures of foods to show the angles and distances to use. Both before and after pictures were included in the instruction manual handed out to the parents. The pictures were then sent via email or short message service (SMS) directly to the research team. In the same email or SMS the parents were also asked to provide information regarding the type of milk, fat (butter, margarine, or oil), meat, bread, cereal, and sauce consumed. When we received the pictures a trained nutritionist (C.D.N.) went through the pictures directly. If the pictures were blurry and difficult to interpret she contacted the parents immediately and asked for clarification. Correspondingly, if information was missing, for example the type of milk consumed she also requested this information. Furthermore, she also contacted the parents if after pictures were missing and requested such pictures. This was rarely the case since almost all parents submitted after pictures with empty plates to confirm that their child had eaten the food in its entirety. In response to this request a few parents submitted a text comment to confirm that everything in the before picture had been eaten and this information was also accepted. All parents participating in the study had their own smart phones. The majority of the days provided were weekend days (68%), which were expected as the MINISTOP trial targets the home environment.

A trained nutritionist (C.D.N.) reviewed all of the food pictures and calculated the EI and the amount of fruits, vegetables, fruit juice, sweetened beverages, candy, ice cream, and bakery products consumed each day. These foods and beverages were selected because they were considered to be relevant markers for healthy (fruits, vegetables, and fruit juice) and unhealthy (sweetened beverages, candy, ice cream, and bakery products) dietary habits; important for obesity prevention interventions. To accurately estimate each portion size, a team of nutritionists took reference pictures of commonly consumed foods in different amounts in or on the standardized china in order to correctly identify the amounts in the provided pictures. The compendium of reference pictures was compiled before the MINISTOP trial was initiated. For whole fruits and some bakery products standardized weights provided by the Swedish Food Agency [24] were used. For each food consumed, the total amount was estimated as the difference between the before and after pictures with extra portions being taken into account when provided. EI was calculated from the intakes of foods and beverages through linkage to the Swedish Food Database [25]. The grams per day of the food categories stated above were then summarized. For each child the mean of the provided days for both EI (kJ/24 h) and the food categories (g/day) were used in the analyses.

Even though all nutritionists working within the MINISTOP trial were trained the same way, there can be differences in how the food pictures are perceived. To evaluate the accuracy of the analysis of the food pictures a second nutritionist (C.A.) evaluated the 39 children’s food pictures. Inter-rater reliability was high, with correlations ranging from 0.893 to 0.957 (*p* < 0.001) for all the food groups. Intra-class correlation coefficients were also strong, ranging between 0.897 and 0.977 for nutritionist one and between 0.941 and 0.986 for nutritionist two.

### 2.4. The 24 h Dietary Recalls

A trained nutritionist (M.L.), different than the one who analyzed the food pictures conducted and analyzed four telephone 24 h dietary recalls [26] with one parent for each child. The days used in the 24 h dietary recalls were the same days that the parents took food pictures. The time and date of the call was scheduled with the parents when they were at the hospital for the measurement. At the beginning of each interview the parents were instructed not to look at the food pictures. Information on the type of food products used in mixed dishes as well as the cooking methods were recorded. Portion sizes were reported by the parent using household measurements (deciliters, tablespoons, or teaspoons). To describe other products such as bread or candy words such as slices or pieces were used. At the end of every meal and at the conclusion of the interview the parent was asked to reflect back to make sure that he/she did not forget any food or beverages consumed by their child. Each interview lasted between 15 to 25 min. The reported intakes were converted into grams using the standardized weights for commonly consumed foods provided by the Swedish Food Agency [24]. EI was calculated from the intakes of foods and beverages through linkage to the Swedish Food Database [25]. The grams of foods and beverages consumed were then summarized. The mean of the provided days for each child for both EI (kJ/24 h) and the food categories (g/day) were used in the analyses.

### 2.5. Statistical Analysis

Values are given as means and standard deviations (SD). Significant differences between mean values were identified using the paired samples *t*-test for parametric data (EI and TEE) and the Wilcoxon Signed Rank test for non-parametric data (food categories). The Bland and Altman method [27] was used to compare EI determined using TECH to TEE measured using DLW. Using this method EI minus TEE (y-axis) was plotted against the average of EI and TEE (x-axis). The mean difference as well as the limits of agreement (±2SD) were then calculated. To test for a trend within methods a linear regression model was fitted between the *x* and *y* axis. The same method was used to compare the intakes of the eight food categories calculated by TECH and the 24 h dietary recalls. Depending on the variables either Pearson or Spearman correlation analyses were conducted to assess the relationship between the variables. In a supplementary analysis we investigated whether the accuracy of the reported energy and food group intake differed by group allocation (intervention or control group). This was done by re-running the above analyses separately for both the intervention and control group and using an un-paired *t*-test. Statistical significance (two-sided) was set at 5%. All analyses were performed with SPSS version 22 (IBM, Armonk, NY, USA).

### 2.6. Power Considerations and Dimensions of the Study

As previously reported, the MINISTOP trial was dimensioned to provide sufficient power to evaluate if the intervention is effective [6]. We also considered power for this validation study within the MINISTOP trial. As recommended when comparing methods we applied the Bland and Altman procedure [27]. Since this procedure does not address a specific hypothesis, no power calculation can be made. It is relevant to note that the original description of this procedure [27] was based on an example with 17 observations. For the comparison of mean values (e.g., EI *versus* TEE) we assumed that TEE was on average 6000 ± 700 kJ, assuming a correlation between EI and TEE of 0.6. Thus, 40 children would provide us with more than 80% power to detect a 5% difference in EI and TEE.

## 3. Results

The 39 participating children were comprised of 22 boys and 17 girls with a mean age of 5.5 ± 0.5 years. The children had a mean: weight of 20.5 ± 4.3 kg, height of 114.3 ± 4.5 cm, and a BMI of 15.6 ± 2.3 kg/m^2^. One child was classified as overweight and two as obese [28]. Mothers had a mean age of 36.3 ± 4.3 years and a BMI of 24.0 ± 3.6 kg/m^2^. The fathers’ average age and BMI was 38.1 ± 5.0 years and 25.4 ± 3.6 kg/m^2^, respectively. Twenty-eight mothers and 25 fathers had a university education. The mean change in body weight from day one to day fourteen was 0.07 ± 0.32 kg.

**Figure 1:**
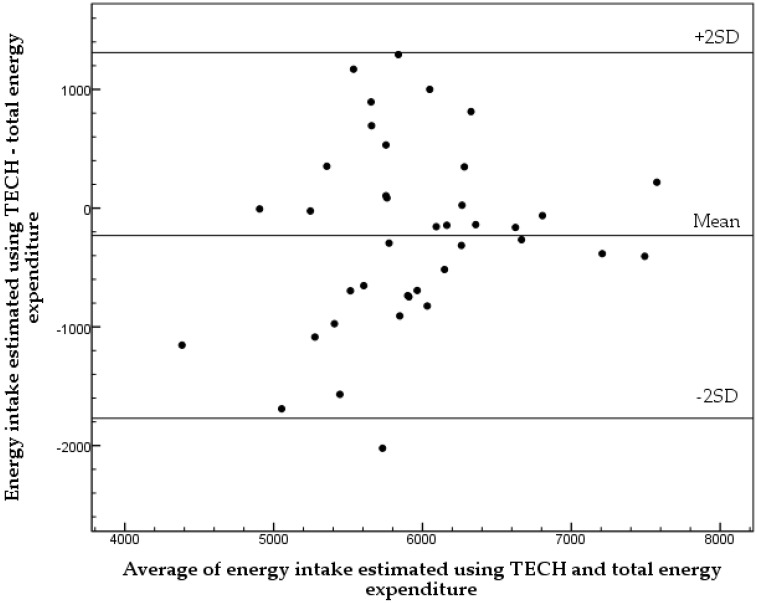
A Bland and Altman plot comparing energy intake using Tool for Energy Balance in Children (TECH) and total energy expenditure measured using the doubly labelled water method in 39 healthy 5.5 year old children. The mean difference between the methods was −220 kJ/24 h with limits of agreement (2SD) of 1540 kJ/24 h. The regression equation was *y* = 0.253*x* − 1733 (*r* = 0.215, *p* = 0.189).

The average EI calculated using TECH was not statistically different (*p* = 0.064) from TEE assessed using DLW (5820 ± 820 kJ/24 h and 6040 ± 680 kJ/24 h, respectively). Figure 1 displays the Bland and Altman plot for EI assessed using TECH compared to TEE. The limits of agreement were wide, and no significant association was found between the average and difference of EI and TEE (*y* = 0.253*x* − 1733; *r* = 0.215, *p* = 0.189). Thus, no trend for that the difference between EI and TEE varied across the different levels of EI was observed, *i.e.*, no systematic bias was found.

Table 1 displays the results for the mean intakes of the eight food categories assessed using TECH and 24 h dietary recalls. No significant differences in the mean values for the eight food categories using TECH and 24 h dietary recalls were found. Table 2 shows the correlations between TECH and the 24 h dietary recalls for all eight food categories. Significant correlations ranging from 0.665 to 0.896 (*p* < 0.001) were found for all categories. When comparisons were conducted between TECH and the 24 h dietary recalls using the Bland and Altman method (see Table S1) all categories had wide limits of agreement and no trends were found except for sweetened beverages. Here a significant correlation was found between the difference and the average of the amount of sweetened beverages consumed between TECH and the 24 h dietary recalls (*ρ* = −0.333, *p* = 0.038).

The average EI assessed using 24 h dietary recalls was 5990 ± 680 kJ/24 h and not statistically different from TEE (*p* = 0.595). The correlation coefficient between EI using 24 h dietary recalls and TEE was *r* = 0.66 (*p* < 0.001). The regression equation between EI assessed using TECH (*x*) and TEE (*y*) was *y* = 0.41*x* + 3670 (*r* = 0.50; *p* = 0.001).

We found no support for that the accuracy of EI and food groups differed between the intervention (*n* = 19) and the control group (*n* = 20). For instance, EI minus TEE for the intervention group was −80 ± 820 kJ/24 h and this was not statistically different from the corresponding value for the control group (−380 ± 700 kJ/24 h) (*p* = 0.23). When plotting EI minus TEE against the average of the two methods, no trends for a bias were observed for the intervention or the control group (intervention: *r* = 0.16, *p* = 0.50 and control: *r* = 0.30, *p* = 0.20). All eight food categories assessed using TECH and the 24 h recalls were significantly correlated for the intervention as well as the control group. For the intervention group, the Spearman correlation coefficients for the eight food categories ranged between 0.59 and 0.92 and between 0.59 and 0.84 for the control group.

**Table 1:** Mean intake of foods estimated by means of TECH and 24 dietary food recalls (*n* = 39).

	TECH ^1,2^		24 h Recall ^3^		
Food Group	Intake ^4^ (g/Day)	Range (g/Day)	Intake ^4^ (g/Day)	Range (g/Day)	*p* ^5^
Fruit	103 ± 65	0–251	110 ± 76	0–293	0.307
Vegetables	64 ± 49	0–223	67 ± 52	0–196	0.255
Fruit & Vegetables ^6^	230 ± 138	8–594	227 ± 148	0–782	0.655
Fruit Juice	56 ± 73	0–313	46 ± 89	0–488	0.087
Sweetened Beverages	77 ± 93	0–533	90 ± 93	0–467	0.161
Candy	19 ± 22	0–87	15 ± 16	0–63	0.290
Ice Cream	12 ± 19	0–75	11 ± 15	0–53	0.728
Bakery Products	19 ± 14	0–63	18 ± 16	0–59	0.369

^1^ Tool for energy balance in children; ^2^ Number of recorded days using TECH: four days (*n* = 31, 79%), three days (*n* = 7, 18%), and two days (*n* = 1, 3%); ^3^ Number of recorded days using 24 h dietary recall: four days (*n* = 27, 70%), three days (*n* = 6, 15%), two days (*n* = 4, 10%), and one day (*n* = 2, 5%) ^4^ Intake is the average of the days provided by both TECH and the 24 h dietary recalls; ^5^ *p* value for the difference between daily intake estimated from TECH and 24 h recalls using the Wilcoxon signed rank test. ^6^ Fruit & vegetables is the sum of the all fruits, vegetables, and fruit juice consumed.

**Table 2:** Correlation coefficient between average food intake (g/day) estimated by means of TECH and 24 h dietary recalls ^1^ (*n* = 39).

Food Group	*ρ* ^1^	*p*
Fruit	0.874	<0.001
Vegetables	0.871	<0.001
Fruit & Vegetables	0.896	<0.001
Fruit Juice	0.665	<0.001
Sweetened Beverages	0.711	<0.001
Candy	0.744	<0.001
Ice Cream	0.753	<0.001
Bakery Products	0.786	<0.001

^1^ Spearman rank order correlation.

## 4. Discussion

TECH is an easy to use dietary assessment method that allows parents of pre-school aged children to take pictures of their child’s meals and send them to researchers with minimal effort. Besides our pilot study [16], this is the first study to assess EI using mobile phones and compare it to TEE assessed using the DLW method in pre-school children. The mean difference between EI and TEE using TECH and DLW, respectively was small (−220 kJ/24 h, *i.e.*, −4%) and not statistically different. The Bland and Altman plot showed no systematic error in EI assessed using TECH across EI levels. The mean difference observed between EI and TEE is similar to or lower than that of other studies in young children (range −6% to 59%) using traditional dietary methods [20,21,29,30,31,32,33]. This demonstrates TECH’s ability to assess average EI in an unbiased way in our particular setting.

There are no gold standard methods for assessing the intakes of foods and beverages. Therefore, to determine TECH’s ability to assess food groups we compared the intakes of eight different food categories assessed using TECH and compared them to the intakes assessed by 24 h dietary recalls. In all of these food categories, there were no significant differences between the mean values assessed using TECH and the 24 h dietary recalls. No significant trends in the Bland and Altman plots were observed for seven of the eight categories. Even though a weak bias was present for sweetened beverages, it is reasonable to conclude that TECH has the ability to assess average values for the majority of the food groups.

Correlations are not recommended when evaluating methods, however, they are commonly used when comparing dietary assessment methods, so they are therefore provided in this validation. We found stronger correlations between the food groups than other studies in this age group when comparing two established dietary methods [34,35,36]. The higher correlations found in our study are likely due to the use of dietary recalls compared to the use of food frequency questionnaires in the other studies. The 24 h dietary recalls covered the same days as TECH, while a food frequency questionnaire captures eating patterns over a longer period of time. Another contributing factor to the high correlations is that the errors between TECH and 24 h dietary recalls are likely to be strongly correlated. The strong correlations found demonstrate TECH’s ability to rank children according to their intake of healthy and unhealthy foods.

As expected, the wide limits of agreement in the Bland and Altman plot demonstrate that TECH has a limited ability to assess EI for individuals. Wide limits of agreement for EI have also been observed in other studies using established dietary methods in children [20,21,29,30,31,32] as well as in adults [37]. Our results were expected as there is a larger day to day variation in EI compared to TEE [38]. Furthermore, wide limits of agreement were observed for all investigated foods. This is also in accordance with previous studies [34].

We previously conducted a pilot study to compare EI using TECH to TEE from DLW in 30 Swedish three year old children [16]. In the current validation study we found smaller limits of agreement and no bias in the Bland and Altman plot. In the pilot study there was a bias to overestimate high energy intakes and underestimate low energy intakes (*r* = 0.73, *p* < 0.001). As a possible explanation, we suggested that the observed bias could be due to only having one day of food recordings [16]. The results obtained in the current study support this suggestion.

As described above TECH was developed for the MINISTOP trial. The MINISTOP trial targets the home environment and therefore, TECH was developed to assess the intake of energy and certain foods at home. In Sweden, the majority of both parents work full-time when they have a child of this age. This means that dietary recordings for the MINISTOP trial as well as this validation study includes mostly weekend days since those are the only days when the parents spend 24 h periods with their child. It may be argued that the agreement between EI using TECH and TEE is affected by the fact that the parents chose recording days and mostly weekend days were covered if the children’s EIs were different between weekdays and weekend days. We cannot exclude that this is the case; however, we find it unlikely since previous reports in Swedish children have shown that even though their intakes of unhealthy foods are higher during weekends, no differences in EI between weekdays and weekend days have been observed [39,40,41].

A major strength of this study was the comparison of EI to TEE assessed using DLW. The DLW method is considered the gold standard for assessing TEE and is used for validating energy intakes [18]. The DLW method can be used to validate EI since the EI should equal the TEE for subjects in energy balance during the measurement period. This criteria was fulfilled in our growing children since they were weight stable throughout the measurement period and the energy content of retained tissue corresponds to approximately 1% of the energy intake at this age [42,43]. Furthermore, this study is also strengthened by the fact that we investigated whether the accuracy of reported EI and the food groups differed by group allocation (intervention *versus* control). Although our data did not provide any evidence that such differences exist, it is important to stress that these comparisons should be interpreted with some caution due to the limited number of children in each group.

Our study also has limitations. Firstly, the argument can be made that the difference in the measurement periods, four days for EI and 14 days for TEE could have affected our results. We find this very unlikely because the day to day variation in TEE is small [44,45]. Furthermore, we were unable to obtain four days of recordings for all of our children, however, when we repeated our analyses including data from only the children with four days of recordings, our conclusions remained the same. We did not want to increase the burden on our parents so we therefore chose the same measurement period for both dietary assessment measures as well as had announced telephone recalls. We cannot exclude that these above mentioned factors produced a better agreement with the 24 h dietary recalls. However, it is important to note that TECH also produced values for EI which were comparable to TEE assessed using the DLW method and this method is not influenced by such factors. Additionally, the relatively small sample size (*n* = 39) and the fact that our parents represented a well-educated and motivated group could limit the generalizability of our results. However, without such parents it may not be at all possible to conduct studies assessing dietary intake in pre-school children. The children in this validation study were representative of the MINISTOP trial in terms of age, weight, BMI as well as parental age, BMI and education. Additionally, the children’s TEE was comparable to previous data in Western children [31,46] and their body size was comparable to Swedish reference data [47]. The average and variation in BMI of the participating parent’s was also in accordance with the Swedish population [48]. However, as common in research, 79% of mothers and 64% of fathers had a university education which is higher than the general Swedish population (52% and 39%, respectively) [49]. Therefore, more studies in parents with a lower educational background need to be performed.

The results of this study demonstrate that TECH can provide unbiased and accurate mean intakes for energy and selected foods. TECH may therefore be a useful tool for intervention studies, such as obesity prevention trials where group comparisons are made. TECH’s ranking ability was also quite good which indicates that it might be useful for large-scale epidemiological studies where energy and food intake levels (e.g., quartiles) are compared. Indeed, TECH showed poor accuracy for individual children, however, these results are consistent with traditional dietary methods [20,21,29,30,31,32]. Our results are promising since TECH has the ability to reduce the burden on the participants by allowing them to take pre and post meal pictures and submit them directly via SMS or email. Compared to traditional methods such as food diaries or 24 h dietary recalls taking pictures is much less time consuming. Data processing is also easier as all of the information is already digitalized. Furthermore, researchers have the ability to analyze the pictures soon after they have been received and immediately request complementary information if needed. Currently, some studies are focusing on automated food picture analysis that will further reduce the burden on researchers [13]. However, future studies are needed in order to make these automated analysis systems more accurate.

In conclusion, TECH was able to accurately estimate average EI as well as the mean intakes of fruits, vegetables, fruit juice, candy, ice cream, and bakery products in pre-school children in our particular setting. TECH has the potential to be a useful tool for studies investigating dietary intake, such as obesity prevention trials, in young children. Future studies should evaluate the accuracy of TECH in other populations for instance in older children and adolescents where the responsibility for the reporting is largely on the children and not the parents.

## Supplementary Files

Supplementary File 1

## Abbreviations

The following abbreviations are used in this manuscript:

DLW: Double labelled water
EI: Energy intake
MINISTOP: Mobile-based intervention intended to stop obesity in preschoolers
TECH: Tool for energy balance in children
TEE: Total energy expenditure
