# Supplementary material for: A Mobile Phone Based Method to Assess Energy and Food Intake in Young Children: A Validation Study against the Doubly Labelled Water Method and 24 h Dietary Recalls
Source: Nutrients. 2016 Jan 15;8(1):50. doi: 10.3390/nu8010050 (PMC4728662; doi:10.3390/nu8010050)
Supplement: Supplementary File 1 [file nutrients-08-00050-s001.docx]

**Supplementary Materials: A Mobile Phone Based Method to Assess Energy and Food Intake in Young Children: A Validation Study against the Doubly Labelled Water Method and 24 h Dietary Recalls**

Christine Delisle Nyström, Elisabet Forsum, Hanna Henriksson, Ylva Trolle-Lagerros,
Christel Larsson, Ralph Maddison, Toomas Timpka and Marie Löf

**Table S1**. Comparisons of intakes of foods and beverages between TECH ^1^ and 24 h dietary recalls in accordance with the procedure by Bland and Altman [27] (*n* = 39).

| **Food Group** | **Mean Difference ^2^ (g/Day)** | **2SD ^3^** | ***ρ* ^4^** | ***p*** |
| --- | --- | --- | --- | --- |
| Fruit | −5.8 | 77.2 | −0.263 | 0.105 |
| Vegetables | −2.9 | 46.1 | −0.247 | 0.130 |
| Fruit & Vegetables | 3.0 | 139.9 | −0.020 | 0.905 |
| Fruit Juice | 11.7 | 111.2 | 0.246 | 0.130 |
| Sweetened Beverages | −12.0 | 107 | −0.333 ^5^ | 0.038 |
| Candy | 4.1 | 29.6 | 0.271 | 0.096 |
| Ice Cream | 1.0 | 28.2 | −0.064 | 0.698 |
| Bakery Products | 1.4 | 19.0 | −0.220 | 0.179 |

^1^ Tool for energy balance in children; ^2^ Calculated as TECH—24 h dietary recall; ^3^ Calculated as twice the standard deviation; ^4^ Spearman rank order correlation; ^5^ The regression equation was *y* = −0.26*x* + 0.044.
